# Benign breast tumors may arise on different immunological backgrounds

**DOI:** 10.1002/1878-0261.13655

**Published:** 2024-05-16

**Authors:** Lilly Anne Torland, Xiaoran Lai, Surendra Kumar, Margit H. Riis, Jürgen Geisler, Torben Lüders, Xavier Tekpli, Vessela Kristensen, Kristine Sahlberg, Andliena Tahiri

**Affiliations:** ^1^ Department of Clinical Molecular Biology (EpiGen) Akershus University Hospital Lørenskog Norway; ^2^ Institute of Clinical Medicine, Faculty of Medicine University of Oslo Norway; ^3^ Department of Research and Innovation Vestre Viken HF, Drammen Hospital Norway; ^4^ Oslo Centre for Biostatistics and Epidemiology, Faculty of Medicine University of Oslo Norway; ^5^ Department of Ocean Sciences Memorial University of Newfoundland St. John's Canada; ^6^ Department of Breast and Endocrine Surgery, Clinic of Cancer Oslo University Hospital Norway; ^7^ Department of Oncology Akershus University Hospital Lørenskog Norway; ^8^ Department of Medical Genetics Oslo University Hospital Oslo Norway

**Keywords:** benign breast tumors, breast cancer, immunity, pathways

## Abstract

Benign breast tumors are a nonthreatening condition defined as abnormal cell growth within the breast without the ability to invade nearby tissue. However, benign lesions hold valuable biological information that can lead us toward better understanding of tumor biology. In this study, we have used two pathway analysis algorithms, Pathifier and gene set variation analysis (GSVA), to identify biological differences between normal breast tissue, benign tumors and malignant tumors in our clinical dataset. Our results revealed that one‐third of all pathways that were significantly different between benign and malignant tumors were immune‐related pathways, and 227 of them were validated by both methods and in the METABRIC dataset. Furthermore, five of these pathways (all including genes involved in cytokine and interferon signaling) were related to overall survival in cancer patients in both datasets. The cellular moieties that contribute to immune differences in malignant and benign tumors were analyzed using the deconvolution tool, CIBERSORT. The results showed that levels of some immune cells were specifically higher in benign than in malignant tumors, and this was especially the case for resting dendritic cells and follicular T‐helper cells. Understanding the distinct immune profiles of benign and malignant breast tumors may aid in developing noninvasive diagnostic methods to differentiate between them in the future.

## Graphical abstract

Benign breast tumors lack invasive potential, offering insights into tumor biology. Our study, using pathway analysis, identified immune‐related pathways distinguishing benign from malignant tumors. Five pathways linked to cytokine and interferon signaling correlated with overall survival. Deconvolution analysis using CIBERSORT revealed higher levels of certain immune cells in benign tumors than in malignant ones.

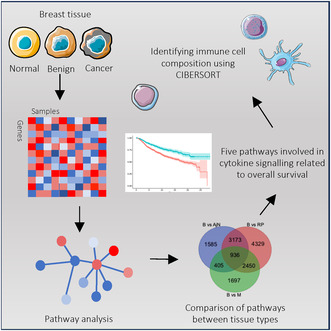

**Abbreviations**
AHUS: Akershus University Hospital
AN: adjacent normal
BMI: body mass index
DC: dendritic cells
ER: estrogen receptor
FDR: false discovery rate
FGFR: fibroblast growth factor receptor
fTh: follicular T‐helper
GSVA: gene set enrichment analysis
IL1B: interleukin 1 beta
LM22: leukocyte signature matrix 22
METABRIC: Molecular Taxonomy of Breast Cancer International Consortium
miRNAs: microRNAs
mRNA: messenger ribonucleic acid
MSigDB: molecular signatures database
OS: overall survival
PDS: pathway deregulation score
PR: progesterone receptor
ROC: receiver operating characteristic
RPs: reduction mammoplasties
ward.D2: Ward's agglomerative linkage method

## 1 Introduction

The composition of breast tissue is heterogeneous, containing different cell types and molecules both within the breast epithelium and the surrounding tissue, that is, the stroma. Previous studies involving gene expression profiling of breast tumors have allowed the identification of different subtypes with different clinical outcomes and responses to therapy [1, 2, 3, 4]. Most of the breast cancer research has focused on understanding and identifying differences between malignant and normal cells, and stroma surrounding tumors, leaving benign tumors somewhat understudied. Nonetheless, previous research has suggested that women diagnosed with benign breast tumors have an increased risk of developing breast cancer later in life [5, 6, 7], although this is still under debate. Benign breast tumors include nonproliferative, proliferative, and proliferative lesions with atypia, and particularly those with proliferative or atypical lesions are associated with increased risk of breast cancer [5, 7]. Abnormal cell growth occurs already at benign stages, and relevant information about molecular changes at early stages of tumor formation may give clarity on mechanisms involved in tumor development. Some of the questions we might want to answer is, once proliferation starts, what prevents the tumor from transforming into malignant tumors and staying benign? In the literature, there is little information available of the molecular changes in benign tumors, even though most lesions that occur in the breast are benign [7].

Previously, we have shown that deregulation of cancer‐related microRNAs (miRNAs) is evident in benign lesions of the breast such as fibroadenomas and fibroadenomatosis in our dataset [8]. A set of miRNAs were identified as deregulated in both benign and malignant tumor tissue compared with normal tissue, with many of these miRNAs previously thought to be mostly cancer related. We showed that the level of deregulation in benign tumors was less than observed in cancer tissue; nonetheless, it stated that similar processes are in place already at early stages of tumor formation [8]. Vidal et al. [9] analyzed the gene expression of fibroepithelial tumors such as fibroadenomas and phyllodes tumors of the breast and classified these into PAM50 subtypes based on gene‐expression data, suggesting that gene expression profiles in benign tumors may provide useful prognostic information. Whereas most of the fibroepithelial tumors were classified as normal‐like subtypes, some of them were classified as Claudin‐low or Basal‐like, indicating there are important differences even within benign tumors.

In this study, we performed pathway analysis on gene expression data from normal breast tissue, benign tumors, and malignant tumors and their corresponding adjacent normal tissue. Two different pathway analysis algorithms, Pathifier [10] and gene set variation analysis (GSVA) [11], were used to identify differences in pathway dysregulation in a clinical dataset. Pathifier is a supervised method that generates a score to each pathway by measuring the deviation of each sample from a baseline condition (normal breast tissue from reduction mammoplasties) [10]. GSVA computes pathway scores unsupervised without taking into account to a sample baseline and utilizes instead density estimates [11]. Although many pathway‐based models have been used to study tumors physiopathology [12, 13, 14], none have to our knowledge looked at the differences between benign and malignant tumors, pathway‐wise. As benign tumors do not possess the ability to invade or metastasize, it is important to identify pathways that are responsible for that. Our aim was therefore to use pathway analysis to identify changes that occur in benign tumors compared to malignant tumors and normal tissue as pathways depicts the larger picture of molecular changes in tumors compared with gene expression alone.

## 2 Materials and methods

### 2.1 Sample and data sources

As a discovery dataset, we have used mRNA expression data from the Akershus University Hospital (Ahus) dataset, as previously described [8, 15]. The study is approved by regional ethical committee (Norwegian Regional Committees for Medical and Health Research Ethics no. 2013/1945 and 2014/895), and patients have given a written consent for research. All samples were handled in accordance to the guidelines of the Declaration of Helsinki. The dataset includes breast tissue samples from malignant (M) tumors (*n* = 210), adjacent normal (AN) tissue (*n* = 239), and core‐needle biopsies from benign (B) tumors (*n* = 12). These samples were obtained from patients at Ahus in the period 2002–2017. The benign tumor biopsies were obtained from women diagnosed with either fibroadenoma or fibroadenomatosis. Tumor samples were either obtained through core needle biopsies or during surgery, and placed in RNA later at −80 °C. To obtain the normal counterpart of the breast tumor, a sample from the vicinity of the tumor were excised (at least 2 cm away from tumor tissue). In addition, 45 breast tissue samples from women undergoing reduction mammoplasty (RP) due to cosmetic or health‐related reasons at the Colosseum Clinic in Oslo were included (REC no. 429‐04148). The clinicopathological data for the whole Ahus dataset is presented for the first time (Table 1). Summary of the clinical information of 506 patients included in this study is shown in Table 1 (for more details, see Table S1).

**Table 1:** Clinical information Akershus dataset. Patient characteristics from benign, malignant, and normal tissue from Ahershus dataset.

	Malignant (*n* = 210)	Adjacent normal (*n* = 239)^a^	Benign (*n* = 12)	Reduction mammoplasty (*n* = 45)
Mean age	62	62	64	40
Histgrade				
I	17	20		
II	89	112		
III	83	100		
No information	21	7		
Tumor grade				
T1	58	72		
T2	120	144		
T3	10	14		
T4	1	2		
No information	21	7		
Lymph node				
N0				
N1				
N2				
ER status				
ER+	147	179		
ER−	43	55		
No information	20	5		
PR status				
PR+	122	147		
PR−	68	86		
No information	20	6		
HER2 status				
HER2+	31	37		
HER2−	140	177		
No information	39	25		
Metastasis				
Yes	16	16		
No	173	216		
No information	21	7		
Current status				
Dead	71	87		
Alive	121	148		
No information	19	4		
PAM50				
LumA	53			
LumB	52			
HER2	25			
Basal	24			
Normal	38			
No information	19	239		

a The adjacent normal tissue is obtained from patients with diagnosed with breast cancer, and the clinical information for these patients are therefore based on the clinical data from the cancer patients.

The METABRIC (Molecular Taxonomy of Breast Cancer International Consortium) cohort [16] was used for validation. We have included mRNA expression data from malignant tumor tissue (*n* = 1939), benign tumor tissue (*n* = 8), and normal breast tissue (*n* = 111) in our analysis. Normal breast tissue in this case is normal tissue adjacent to the tumor tissue.

### 2.2 Gene expression analysis

For the Ahus dataset, mRNA levels were measured using Agilent Technologies (Santa Clara USA) Human Gene Expression 4 × 44k and 8 × 60k microarrays, as previously described [8, 15]. A total of 16 024 mRNA expression profiles from 506 samples from the Ahus dataset was used as input for Pathifier and GSVA pathway analysis tools. During normalization, nonuniform spots were excluded, and missing data were imputed using local least squares imputation. All data were log_2_‐transformed and quantile‐normalized. To have a single expression value per gene per sample, the values corresponding to probes with identical Gene Entrez ID were averaged.

For METABRIC, all the samples were run on the Illumina HumanHT‐12 v3 platform and data were normalized, as previously described [16]. A total of 18 269 mRNA expression profiles from 2135 samples were used as input for pathway analysis.

### 2.3 Pathway gene sets and analysis

**Fig. 1:**
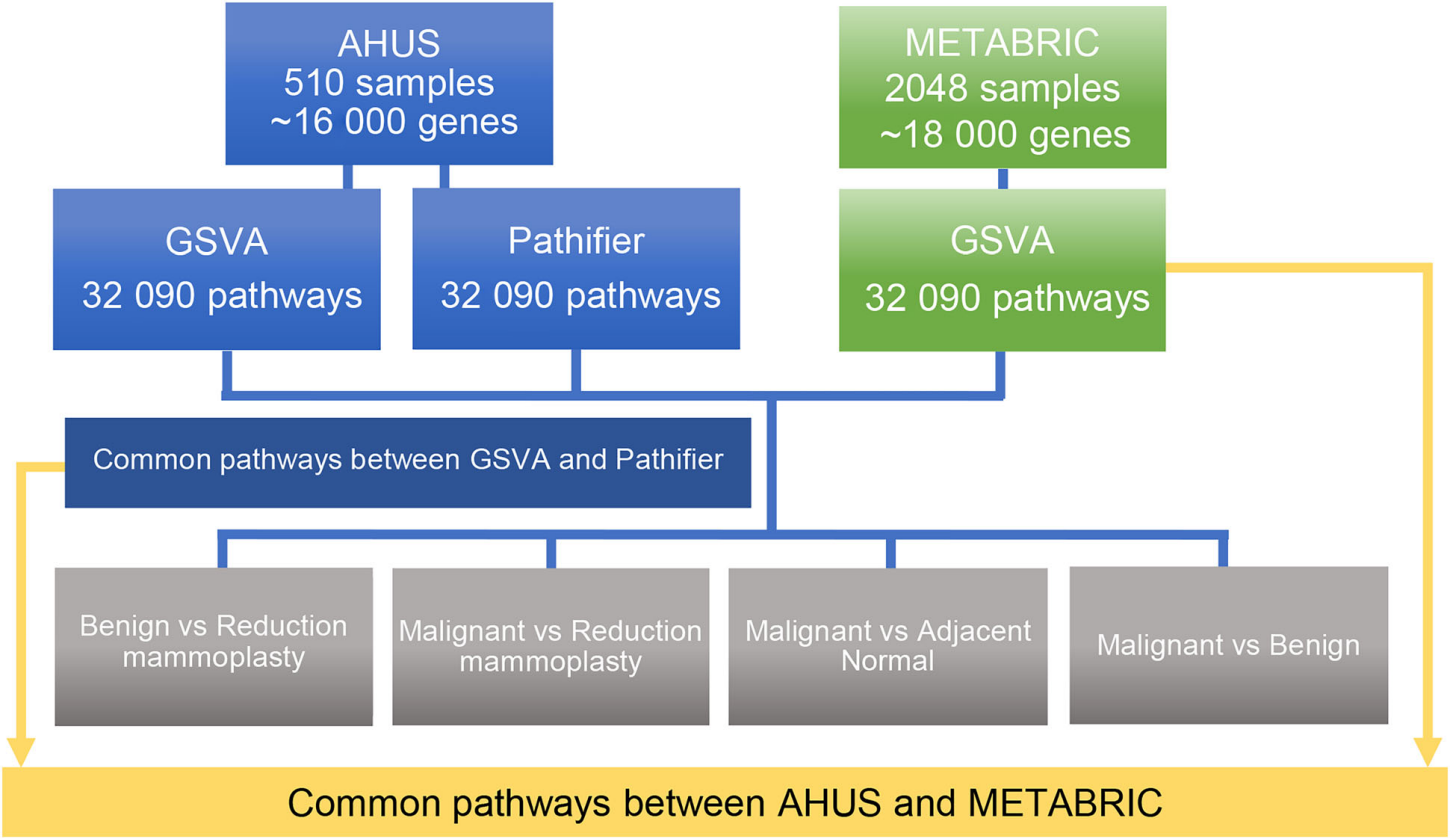
Analysis workflow and dataset utilization. Flowchart of the analysis performed in this study using the Akershus University Hospital (Ahus) dataset as discovery dataset and Molecular Taxonomy of Breast Cancer International Consortium (METABRIC) dataset for validation. For the Ahus dataset, both Gene Set Variation Analysis (GSVA) and Pathifier were used. For the METABRIC dataset, we only used GSVA results for validation.

In total, 32 090 gene sets were downloaded from the Molecular Signatures Database (MSigDB version 7.4) and used for pathway analysis. The gene sets included curated gene sets (*n* = 6290), oncogenic signature gene sets (*n* = 189), immunological signature gene sets (*n* = 4872), hallmark gene sets (*n* = 50), positional gene sets (*n* = 278), cell type signature gene sets (*n* = 671), regulatory target gene sets (*n* = 3731), computational gene sets (858), and ontology gene sets (14 998). The collection of gene sets are based on published transcriptomics studies in cells, humans, and mouse models. The name of each gene set is context‐specific with direction of change for each group of genes per experiment. The project flow is described in Fig. 1. Pathway analysis was performed using the two different tools and two datasets, Ahus and METABRIC. The Ahus dataset was used as a discovery dataset, and METABRIC was used for validation.

### 2.4 Pathifier

Pathway deregulation score (PDS) was calculated for each pathway in the Ahus dataset by the Pathifier bioconductor package 1.16.0 in R [10]. Pathifier transforms gene expression quantification into pathway level measurements by comparing the deregulation profiles of selected groups with the pathway profiles from the healthy normal controls, as has previously been described [17]. In this case, we used the RPs as the normal baseline control. Pathway deregulation score ranges from 0 to 1, where 1 is high deregulation and 0 is no deregulation.

### 2.5 Gene set variation analysis (GSVA)

GSVA is a nonparametric method to analyze variation of pathway activity over a sample‐population in an unsupervised manner. GSVA calculates sample‐wise gene‐set enrichment scores as a function of genes inside and outside the gene sets, and estimates variation of gene set enrichment over the samples, independent of any class label. GSVA is an open software package in R (version 4.3) [11] and used for analysis using both the Ahus and METABRIC dataset.

### 2.6 Visualization of gene sets with heatmap

Unsupervised and supervised clustering on Euclidean distance of values obtained from Pathifier and GSVA was constructed using Ward's agglomerative linkage method (ward.D2) and visualized as a heatmap by hierarchical clustering using the pheatmap package in R version 4.3.

### 2.7 Statistical analysis

All statistical analyses were conducted in the R software (ver. 4.3). Between‐group comparisons were performed using two‐sided Student's *t*‐test. To account for the imbalance in sample sizes between the groups, 1000‐fold permutation tests were applied to the *t*‐statistics obtained from each pathway comparison. Permutation tests are nonparametric and involve reshuffling the data points among the groups repeatedly to create a null distribution. By comparing the original *t*‐statistic with this null distribution, a more accurate *P*‐value can be computed, which is especially valuable when dealing with unequal sample sizes. Finally, to control for multiple comparisons and reduce the chances of Type I errors, all *P*‐values were adjusted using the Benjamini–Hochberg false discovery rate (FDR) method. This adjustment is crucial as it ensures that the proportion of false positives among the statistically significant results is controlled, thereby enhancing the reliability and validity of the findings.

A Lasso‐penalized Cox regression model was employed to identify differentially expressed pathways associated with overall survival (OS) using the combined Ahus and METABRIC datasets. The lasso penalty was incorporates into Cox regression to reduce the number of pathways using 10‐fold cross validation based on glmnet package in R. The selected pathways were then used to construct a prognostic pathway signature of the breast cancer patients in the datasets based on a linear combination of the regression coefficients (β) derived from it multiplied with its pathway score. Patients were divided into high‐ and low‐risk groups based on the optimal cutoff of the prognostic gene signature determined based on log‐rank test. Kaplan–Meier analysis, area under the curve (AUC) of the receiver operating characteristic (ROC) curve, Harrell's concordance index, and a calibration plot comparing predicted and observed OS were used to evaluate the performance of the prognostic gene signature.

### 2.8 Deconvolution of immune cells using CIBERSORT

CIBERSORT is a signature‐based method that combines a signature gene matrix with machine learning to identify the composition of immune cells in bulk tissue [18, 19]. In total, 547 genes are used as the signature file (LM22) to distinguish 22 different immune cells in the Ahus and METABRIC dataset. CIBERSORT was run with the following options: absolute mode, LM22 signature gene file, 1000 permutations, and quantile normalization disabled.

### 2.9 Correlation analysis

Spearman correlation analysis was used to calculate correlations between pathways and estimated immune cell abundances using samples from the Ahus dataset. Here, we performed correlation between the output of GSVA for each pathway, and the score from CIBERSORT for each immune cell type using the Ahus dataset. Rho values ranged from −0.3 to 0.4.

## 3 Results

### 3.1 Comparison of performance of Pathifier and GSVA

As a supervised method that generates a deregulation score to each pathway by measuring the deviation of each sample from a baseline condition (normal breast tissue from reduction mammoplasties), Pathifier showed higher sensitivity to observe differences, but is dependent on equally normalized control dataset. GSVA is a more robust but less sensitive approach as it computes pathway scores unsupervised without taking into account to a sample baseline and utilizes instead density estimates. We performed both analyses and the overlapping pathways are shown below, unless specified otherwise.

### 3.2 Identification of pathways specific to benign tumors

Pathifier and GSVA were applied to mRNA expression from the Ahus dataset (*n* = 510) including malignant and benign breast tumors in addition to normal breast tissue from two different sources. A pathway score was generated for all 32 090 pathways across all samples. The different categories of pathways that were identified by both methods as significantly different (*P* < 0.05) between the different tissue types (normal, normal adjacent to tumor, benign and malignant) using both methods are presented in Table 2. The main categories were according to position and function (regulatory target gene sets) as well as biological function (oncogenic signatures, immunological and cell type signatures gene sets) as defined by MigSig database. As expected, most differences were observed between malignant and normal tissue. However, we found that normal tissue adjacent to the tumor (AN) had a very high deregulation score compared to normal tissue from reduction mammoplasty (RPs) and benign tissue (Fig. S1). There is also a high number of significantly different pathways between RP and AN, which indicates a strong field effect of the tumor (Table 2).

**Table 2:** Number of significantly different pathways in tissue types. Number of significantly different pathways in tissue types using both GSVA (unit) and Pathifier (PDS scores) (*P* < 0.05)^a^.

	M vs B	M vs RP	M vs AN	B vs RP	RP vs AN	B vs AN
Common between GSVA and Pathifier	5469	12 681	14 061	6091	10 603	10 887
Hallmark gene set	18 (0%)	32 (0%)	37 (0%)	17 (0%)	27 (0%)	24 (0%)
c1 positional gene set	72 (1%)	162 (1%)	161 (1%)	67 (1%)	141 (1%)	142 (1%)
c2 curated gene sets (*n* = 6366)	1643 (30%)	3795 (30%)	4269 (30%)	1801 (29%)	3096 (29%)	3240 (30%)
c3 regulatory target gene sets (*n* = 3726)	879 (16%)	2148 (17%)	2392 (17%)	1103 (18%)	1872 (18%)	1907 (18%)
c4 computational gene sets (*n* = 858)	185 (4%)	546 (5%)	512 (4%)	222 (4%)	411 (4%)	451 (4%)
c5 ontology gene sets (*n* = 15 473)	1109 (20%)	2533 (20%)	2882 (20%)	1134 (19%)	2378 (22%)	2198 (20%)
c6 oncogenic signature gene sets (*n* = 189)	26 (1%)	124 (1%)	138 (1%)	76 (1%)	53 (1%)	95 (1%)
c7 immunological gene sets (*n* = 5219)	1468 (27%)	3055 (24%)	3464 (25%)	1571 (26%)	2491 (24%)	2667 (24%)
c8 cell type signature gene sets (*n* = 700)	74 (1%)	194 (2%)	218 (2%)	107 (2%)	142 (1%)	163 (2%)

a All *P* values are FDR corrected.

**Fig. 2:**
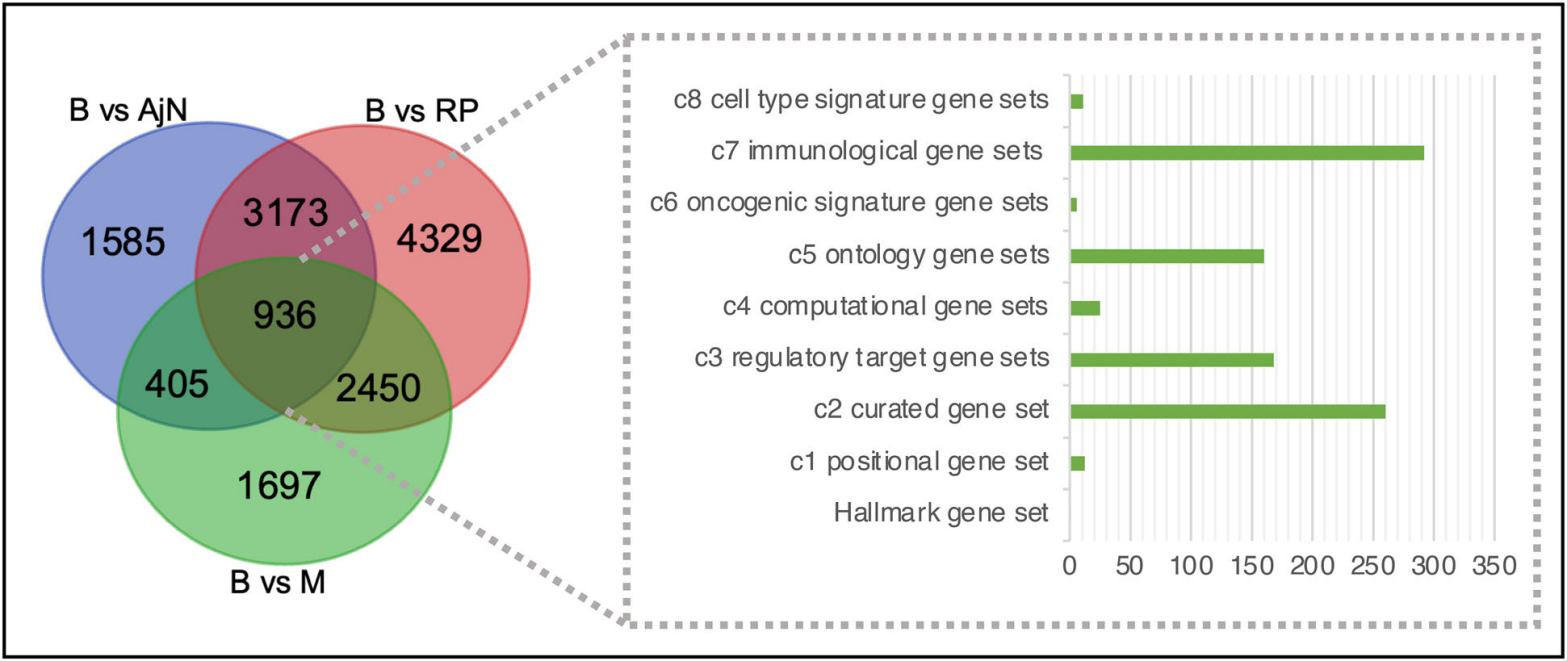
Comparison of significant pathways across tissue types. Venn diagram showing the number of significant pathways that are different between benign (B) and malignant (M) tumors and normal tissue (reduction mammoplasties (PR) and adjacent normal (AjN)). In total, 936 pathways were overlapping in all three comparisons, making them specific to benign tumors. Of these 936 pathways, a large amount of the pathways are found in the immune related gene set, followed by regulatory target gene sets and the curated gene sets.

Benign tumors also showed significant differences when compared to malignant tumors and normal tissue. We identified a total of 18 969 pathways as significantly different (*P* < 0.05) between benign and malignant tumors using Pathifier, and 12 812 pathways identified through GSVA. Through our analysis, 5469 common pathways were identified between benign and malignant tumors in the Ahus dataset, whereas a set of 6091 pathways were commonly different between RP and benign using both GSVA and Pathifier results (Table 2). Several pathways were different between benign, malignant, and normal tissue (Fig. 2), and a total of 936 pathways were identified to be related to only benign tissue. Of these, the largest fraction is the immune related gene set (c7), followed by regulatory target gene sets (c3) and the curated gene sets (c2) (Fig. 2).

**Fig. 3:**
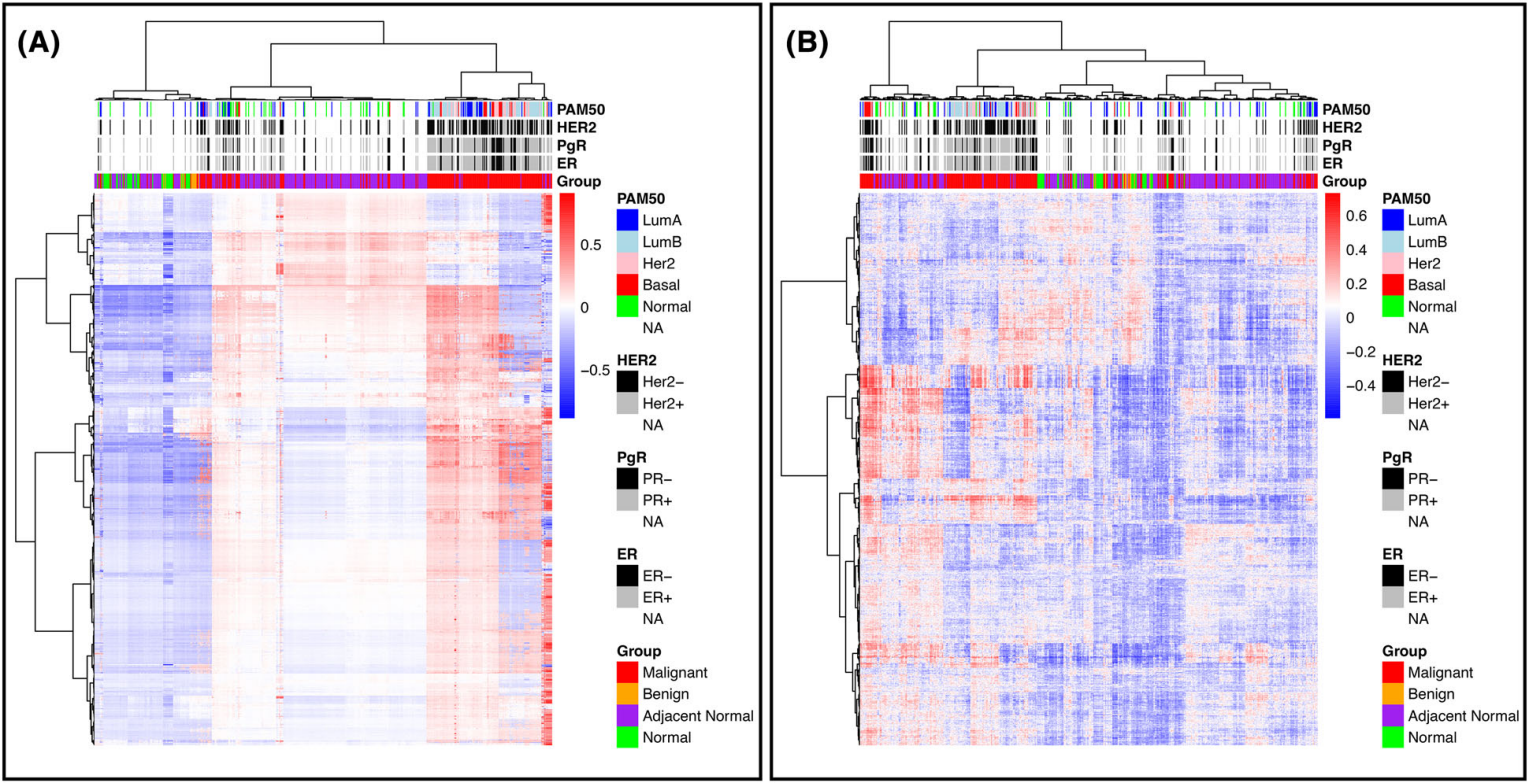
Heatmap of immune‐related pathway differences. Heatmap of 1468 immune related pathways identified as significantly different (*P* < 0.05) between malignant and benign tumors in the Akershus University Hospital dataset. Pathways were identified through both (A) Pathifier and (B) Gene Set Variation Analysis (GSVA). Horizontal axis represents samples, whereas on the vertical axis represents the different pathways. Blue color indicates low pathway score, whereas red indicates high pathway score.

Furthermore, 27% (*n* = 1468) of the gene sets that are significantly different between malignant and benign tumors identified through Pathifier and GSVA are also immune related (c7) (Table S2). A heatmap generated using these 1468 immune‐related pathways, show that the results from Pathifier (Fig. 3A) and GSVA (Fig. 3B) exhibit similar clustering pattern of samples. Pathifier clusters into three clusters where GSVA cluster into 4. Several of the samples are found to overlap within clusters from both methods. In both heatmaps, all the 12 benign samples are found in cluster one, together with the majority of the RP samples. Cluster two holds most of the malignant tissue samples and cluster three most of the AN samples.

### 3.3 Analysis and validation of immune related pathways in the METABRIC dataset

**Fig. 4:**
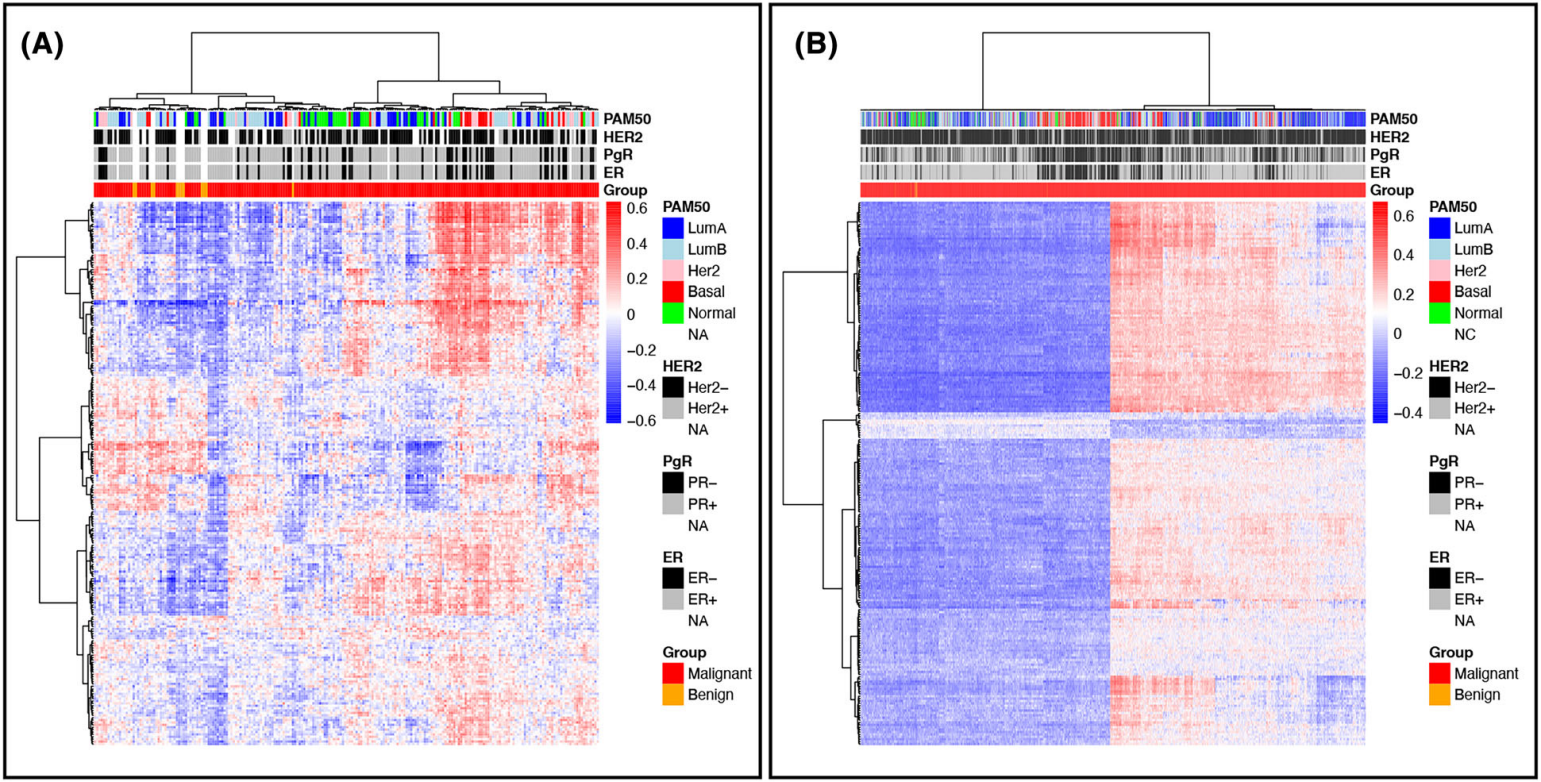
Comparative analysis of immune pathways across datasets. Unsupervised heatmap with 227 immune related pathways (vertical axis). Pathways where identified in (A) Akershus University hospital (Ahus) data set, and (B) Molecular Taxonomy of Breast Cancer International Consortium (METABRIC) dataset. Vertical axis represents pathways, blue color indicates low pathway score, whereas red indicates high pathway score. Horizontal axis represents patient with benign (orange) and malignant tumors (red). Annotation includes PAM50 status (pink = Her2, red = Basal like, light‐blue = Luminal A, blue = Luminal B, green = Normal like), Estrogen receptor status (white positive, black negative) Progesterone receptor status (white positive, black negative) and Human epidermal growth factor 2 status (white positive and black negative) for each patient.

In the METABRIC dataset, 4278 pathways were differentially expressed between malignant and benign tumors. Among these, 639 (15%) were immune‐related pathways (Table S3). Comparing the results from GSVA and Pathifier from the Ahus dataset and GSVA results from the METABRIC dataset, a total of 227 immune related pathways (c7) (Table S4) were identified as commonly significantly different (*P* < 0.05) between benign and malignant tumors, comprising mostly of genes involved in interferon and cytokine signaling (Table S5). A complete overview of all comparisons for the tissue types in the different datasets can be found in the Tables [Link], [Link], [Link], [Link], [Link]. Heatmap generated with the 227 immune‐related pathways clearly separates the malignant tumors from the AN tissue and RPs (Fig. S2). However, the clusters generated with heatmaps of malignant and benign tumors only, shows that the malignant tumors can be divided into two clusters (Fig. 4). One cluster where malignant tumors have high mean score of immune‐related pathways and one that has a “low immune score” (Fig. S3A). The benign samples are in the “immune low” cluster and have a lower mean score compared with the malignant samples (Fig. S3B).

### 3.4 Identification of survival‐related differentially expressed pathways and establishment of a prognostic signature using 227 immune pathways identified in Ahus and METABRIC

**Fig. 5:**
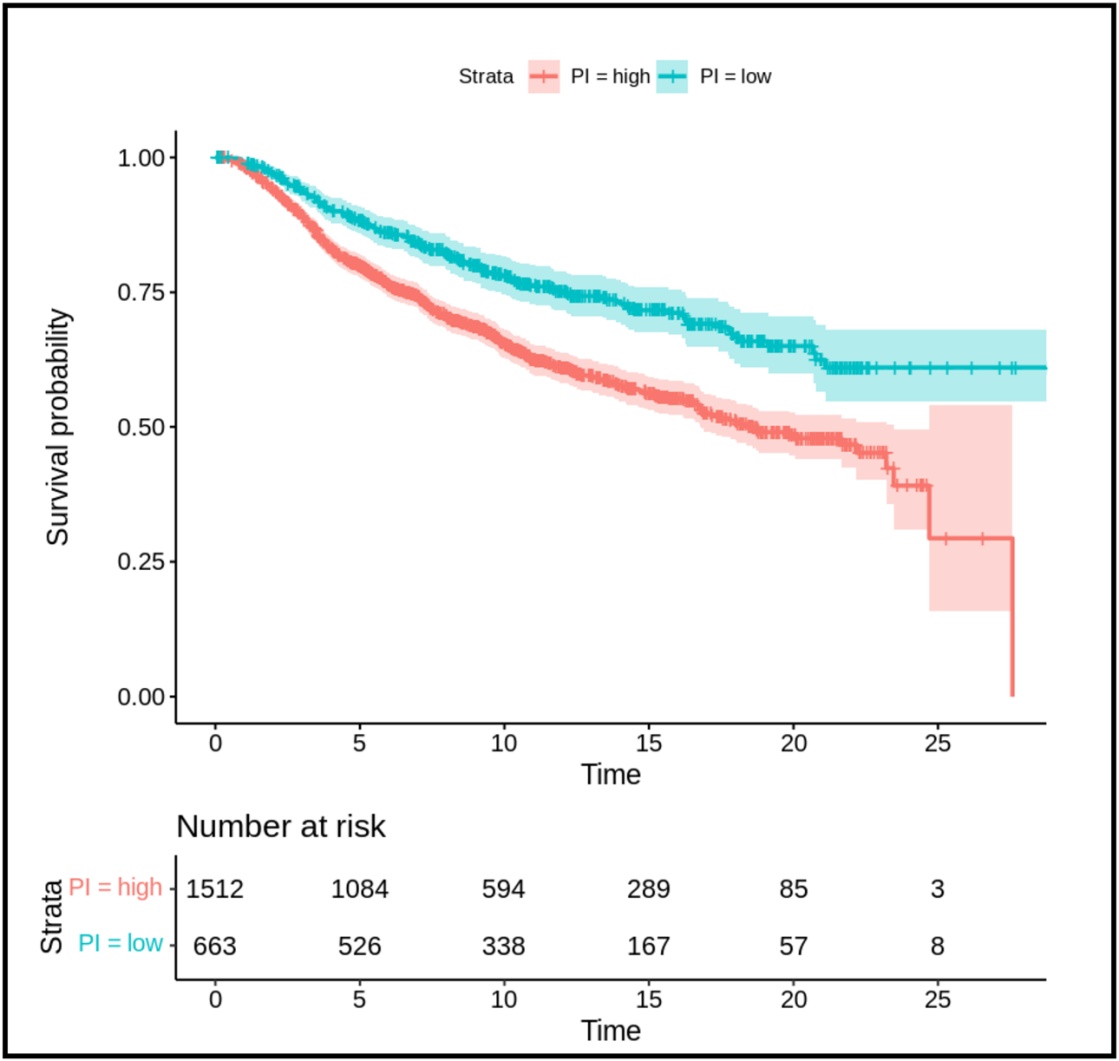
Survival analysis based on pathway risk groups. Kaplain–Meier estimates for the overall survival of breast cancer patients divided into high risk (in red) and low risk (in blue) groups using prognostic index (PI) based on five pathways combined with their respective 95% confidence intervals (indicated by the shaded area). Time, in months, is on the *x*‐axis.

Analysis with 227 immune‐related pathways identified five gene sets (Table 3) that predicts OS in breast cancer patient using both Ahus and METABRIC datasets, based on high or low GSVA score (Fig. 5). One gene set (GSE29614) predicts better survival with high mean GSVA score in malignant tumors, whereas four gene sets (GSE18791, GSE30083, GSE360, and GSE18281) predicts worse survival with low mean GSVA score.

**Table 3:** Impact of immune‐related gene sets on overall survival. Hazard ratios and 95% confidence intervals (CI) from Cox regression analysis of immune related gene sets predicted to have an impact on overall survival using both Ahus and METABRIC dataset.

Gene set/pathway	Genes in pathway (*n*)	Genes sign. different between M and B Ahus (*n*)	Hazard ratios	Lower 95% CI	Upper 95% CI
GSE18791_CTRL_VS_NEWCASTLE_VIRUS_DC_1H_UP	181	17	0.1954831	0.08576932	0.4455397
GSE29614_DAY3_VS_DAY7_TIV_FLU_VACCINE_PBMC_UP	187	17	2.4365155	1.0599577	5.600797
GSE30083_SP2_VS_SP4_THYMOCYTE_UP	200	20	0.3505122	0.15323896	0.8017467
GSE360_DC_VS_MAC_L_MAJOR_UP	200	37	0.4841388	0.22924269	1.0224554
GSE18281_SUBCAPSULAR_CORTICAL_REGION_VS_WHOLE_CORTEX_THYMUS_DN	198	26	0.41879820	0.160 403 261	1.0934438

Using the gene expression omnibus, we characterize the pathway function of the five gene sets described above. GSE29614, which was the only gene set associated with positive OS, is a pathway involved in initiating an adaptive immune response after flu vaccination. GSE18791 gene sets are involved in an antiviral response. GSE30083 gene set that is described in being involved in thymus maturation of T cells in mice. GSE360 gene set is involved in macrophage and dendritic response to pathogen, whereas GSE18281 gene set is involved in Thymic T‐cell differentiation. Furthermore, we analyzed the genes within each of the five gene sets using Reactome Pathway browser (Table S11). Collectively, the genes within these pathways are part of interferon and cytokine signaling (Table S12). GSE29614, the only gene set associated with better OS in malignant tumors, include genes involved in signaling pathways, especially fibroblast growth factor receptor (FGFR) signaling (Table S13).

### 3.5 Deciphering immune cell types using CIBERSORT

**Fig. 6:**
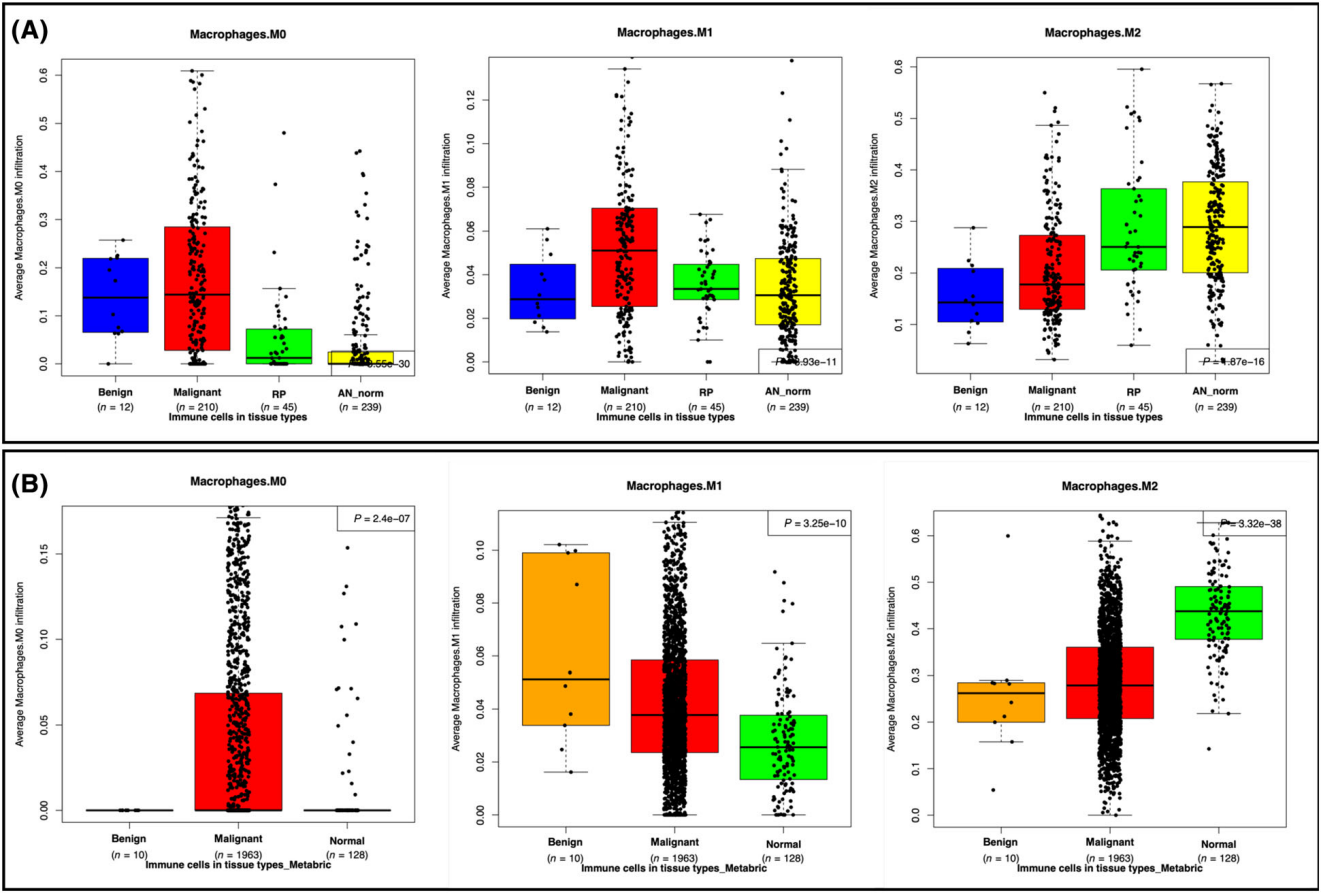
Macrophage expression across tissue types. Boxplot of macrophage expression fore different tissue types. Macrophage expression of tissue types found in the (A) Akershus University Hospital dataset and, (B) Molecular Taxonomy of Breast Cancer International Consortium (METABRIC) dataset.

Since we have previously shown that different immune cell types can influence tumor growth, survival, and sensitivity to therapies [20]. We studied the composition of immune cell types in the different tissue groups based on gene expression using CIBERSORT. The mean expression of 22 immune cells in malignant, benign, and normal breast tissue, and significance values (FDR < 0.05) between different breast tissue types are displayed in Table 4. The results show a significantly higher abundance of resting dendritic cells (DC) and follicular T helper (fTh) in benign tissue. This indicates that these two immune cell types might differentiate benign tumors from normal tissue, but also from malignant tissue. A large proportion of immune cells (*n* = 17) are significantly different (FDR < 0.05) when comparing malignant tumors to adjacent normal tissue. Also, we observe that the adjacent normal tissue is very different to normal tissue from noncancer patients (RPs) when looking at the immune cell composition. Furthermore, macrophages are significantly different in many of the tissue groups analyzed (Fig. 6A). Based on CIBERSORT results, macrophages M0 are more abundant in malignant and benign tumors compared with normal tissue, whereas macrophages M2 are more abundant in normal tissue compared with malignant and benign tumor tissue. Macrophages M1 seem to be highest in malignant tissue compared to benign and normal tissue. Using the METABRIC dataset to identify the differences in cell type composition between the different tissue types, fTh cells did show higher expression in benign tumors compared to malignant and normal tissue but it was not statistically significant (Table S14). Analyzing the macrophage expression, we observe that at least macrophages M2 follow the same trend as in the Ahus dataset, with higher expression in normal tissue compared with benign and malignant tumor tissue (Fig. 6B). When analyzing the different cells in comparison with PAM50 subtypes, we identified that M0 macrophages were highly expressed in benign and malignant tumors (Fig. S4A), but benign tumors were more similar in expression to LumA tumors compared with the other subtypes. M1 macrophages were highly expressed in HER2‐enriched tumors, and very low expression in benign and normal tissue (Fig. S4B). M2 macrophages were still high in normal tissue, but highest in LumA tumors compared with benign and other subtypes (Fig. S4C). We observe that benign tumors had higher fTh and dendritic cell expression compared with the different subtypes and normal tissue altogether (Fig. S4D,E).

**Table 4:** Expression of immune cells across tissue types in Ahus dataset. Immune cells expressed in malignant, benign, adjacent normal and reduction mammoplasty in the Akershus University Hospital dataset. AN, adjacent normal; B, benign; FDR, false discovery rate; M, malignant; NK, natural killer; RP, reduction mammoplasty; T, T cells.

Celltype	Immune branch	Mean B	Mean M	Mean RP	Mean AN	FDR B vs M	FDR B vs RP	FDR M vs RP	FDR M vs AN	FDR RP vs AN
Memory B cells	Adaptive	0.001	0.009	0.007	0.008	2.8E‐01	1.0E+00	**2.2E‐02**	1.4E‐01	1.2E‐01
Naive B cells	Adaptive	0.048	0.037	0.044	0.020	4.3E‐01	9.0E‐01	**4.6E‐02**	**6.5E‐08**	**2.0E‐07**
Plasma cells	Adaptive	0.179	0.113	0.132	0.112	9.2E‐02	3.9E‐01	6.3E‐01	1.2E‐01	2.9E‐01
Memory activated CD4 T	Adaptive	0.026	0.035	0.045	0.012	8.2E‐01	5.2E‐01	1.1E‐01	**4.1E‐09**	**2.8E‐07**
Memory resting CD4 T	Adaptive	0.057	0.061	0.075	0.097	7.5E‐01	5.2E‐01	**4.3E‐02**	**1.0E‐10**	7.5E‐02
Naïve CD4 T	Adaptive	0.063	0.037	0.019	0.016	6.2E‐01	1.1E‐01	**6.0E‐04**	**1.3E‐10**	6.9E‐01
CD8 T	Adaptive	0.027	0.063	0.036	0.046	2.5E‐01	6.9E‐01	7.2E‐02	9.3E‐02	3.1E‐01
Follicular helper T	Adaptive	0.043	0.019	0.023	0.008	**3.9E‐02**	**2.8E‐02**	7.0E‐01	**6.9E‐08**	**1.0E‐02**
Regulatory T	Adaptive	0.011	0.014	0.005	0.004	6.9E‐01	5.6E‐02	**7.0E‐03**	**8.9E‐14**	1.6E‐01
Activated dendritic cells	Innate	0.000	0.001	0.000	0.000	6.2E‐01	8.2E‐01	5.0E‐01	1.6E‐01	2.0E‐01
Resting dendritic cells	Innate	0.004	0.001	0.001	0.001	**2.5E‐03**	**6.2E‐03**	5.4E‐01	8.8E‐02	7.9E‐01
Eosinophils	Innate	0.005	0.002	0.012	0.007	9.1E‐02	1.0E+00	**5.1E‐04**	**1.5E‐02**	6.3E‐02
M0 Macrophages	Innate	0.139	0.176	0.054	0.039	7.5E‐01	**7.6E‐03**	**3.5E‐06**	**5.4E‐28**	**3.6E‐02**
M1 Macrophages	Innate	0.033	0.057	0.035	0.034	1.1E‐01	8.2E‐01	**1.4E‐03**	**4.0E‐11**	3.3E‐01
M2 Macrophages	Innate	0.154	0.207	0.286	0.291	2.5E‐01	**4.7E‐03**	**3.2E‐04**	**7.2E‐15**	5.8E‐01
Activated mast cells	Innate	0.006	0.019	0.010	0.045	1.1E‐01	8.2E‐01	**2.6E‐03**	**2.2E‐05**	**5.4E‐06**
Resting mast cells	Innate	0.070	0.044	0.079	0.057	2.5E‐01	8.2E‐01	**5.1E‐04**	**1.1E‐02**	**3.6E‐02**
Monocytes	Innate	0.037	0.032	0.048	0.082	3.2E‐01	5.2E‐01	**2.3E‐04**	**1.0E‐22**	**1.3E‐03**
Neutrophils	Innate	0.019	0.010	0.029	0.021	2.5E‐01	9.0E‐01	5.5E‐02	**1.1E‐06**	5.8E‐01
Activated NK cells	Innate	0.040	0.043	0.043	0.093	8.2E‐01	8.2E‐01	5.3E‐01	**8.7E‐21**	**2.8E‐07**
Resting NK cells	Innate	0.021	0.008	0.007	0.004	6.9E‐01	5.2E‐01	5.0E‐01	**2.7E‐03**	5.1E‐01
Gamma delta T	Innate	0.016	0.013	0.009	0.003	8.2E‐01	8.2E‐01	7.5E‐01	**7.2E‐12**	**5.4E‐06**

Values in bold are corrected *P*‐values that are statistically significant.

### 3.6 Cellular composition on the five survival‐related immune pathways

**Fig. 7:**
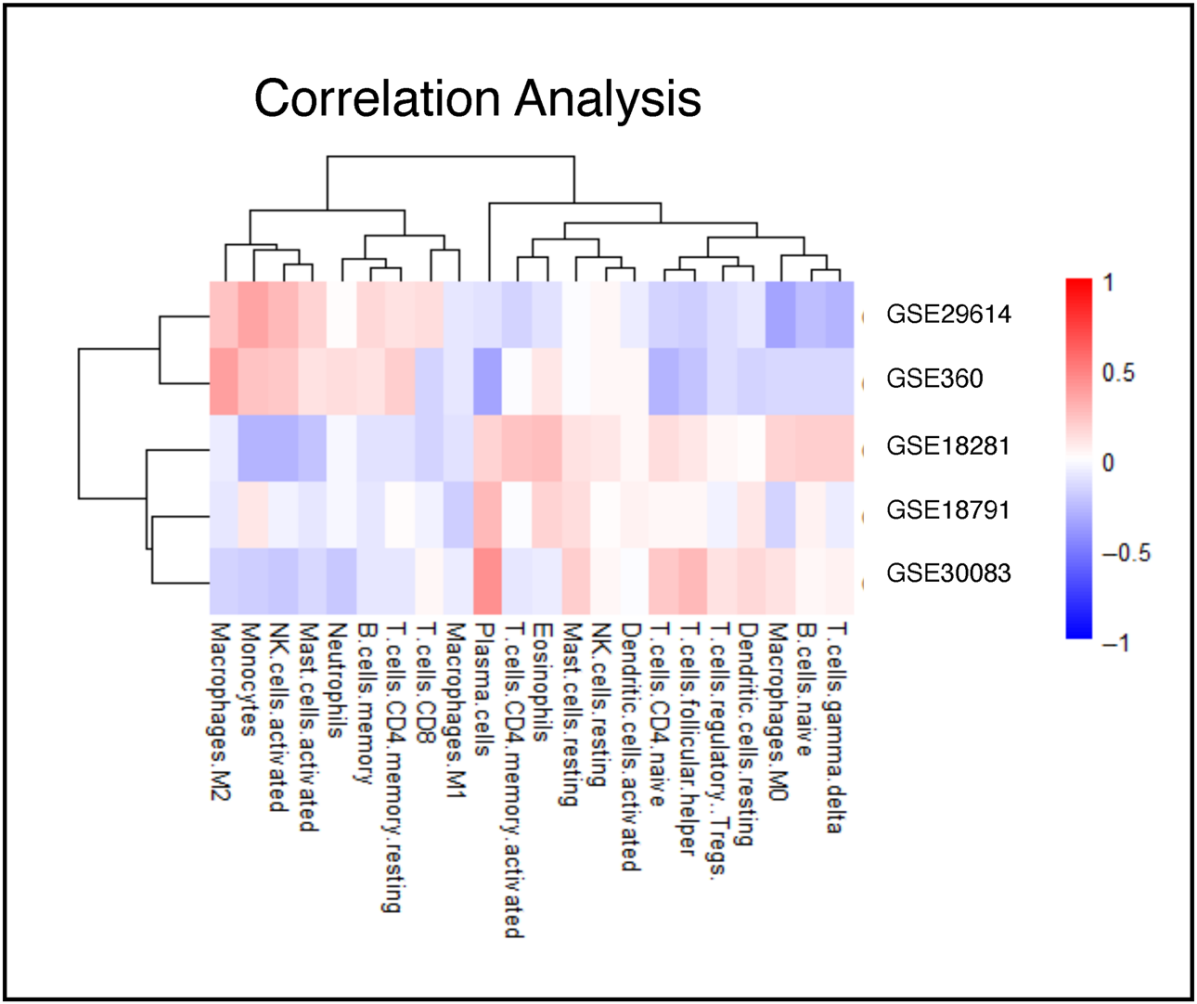
Correlation heatmap of immune cell types and pathways. Heatmap of correlation values (Rho) between immune cell types and five pathways in the Akershus University Hospital dataset. Red indicating positive correlations whereas blue indicates negative correlation.

To further characterize the five immune‐related pathways that predict survival in both datasets we correlated the gene sets to the relative abundance of 22 cell types from CIBERSORT (Fig. 7). Here, we identify that GSE29614 and GSE360 are highly correlated with CD4 memory resting T cells, memory B cells, neutrophils, activated mast cells, activated natural killer cells, monocytes, and M2 macrophages. The three other gene sets were highly correlated with gamma delta T cells, Naive B cells, M0 macrophages, resting and activated DC, Treg cells, fTh cells, CD4 naïve T cells, resting NK cells, CD4 memory activated T cells, and plasma cells.

## 4 Discussion

Benign lesions of the breast are far more frequent than malignant tumors [7]. The increased resolution in which lesions are discovered requires better methods to distinguish benign from malignant, preferably preoperatively. Here, we have attempted to identify molecular differences between malignant and benign tumors using the two most frequently used *in silico* pathway algorithms. We used two different datasets, Ahus [8, 15] as the discovery and METABRIC [16] for validation, both which have been published previously. We first compared two different pathway analytical algorithms, GSVA and Pathifier, in order to find the most significant pathways that are different between benign and malignant tumors using the Ahus dataset. We find 936 pathways to be associated with benign tumors when comparing with other tissue types. Of the 936 pathways, 292 were immune related. Furthermore, the largest differences between benign and malignant tumors were also identified to be immune related, which indicated that immunity plays a major role in differentiating benign and malignant tumors. Based on these results, we performed further analysis using only the c7 gene set list identified as immunological gene sets in MSigDB.

While low levels of estrogen exposure stimulate macrophages and other inflammatory cell populations, very high levels are immune suppressive. In our previous study, using single gene markers (granzyme, IL1B, KRT5), we show that normal tissue adjacent to the tumor is affected by the tumor, influenced by obesity and age, which interact to influence inflammatory cell populations in normal breast tissue [15]. Age and Body Mass Index (BMI) were independently associated with inflammation in adjacent normal tissue but not tumors. Estrogen Receptor (ER)‐negative tumors had elevated macrophage expression compared with matched normal tissue, but ER‐positive tumors showed an unexpected decrease in macrophage expression. We found an inverse relationship between the increase in tumor estrogen pathway expression compared with adjacent normal tissue and tumor macrophage score. We validated this finding in 126 breast tumor‐normal pairs from the previously published METABRIC cohort [15]. These findings are in support of results obtained from Pathifier on the Ahus dataset showed that the PDS score of adjacent normal tissue is higher compared with normal tissue from non‐cancer patients, indicating that deregulation is already occurring in the surrounding stromal environment of the tumor.

The METABRIC dataset [16] was used for validation, as it was the only dataset available with including benign tumors. From our analysis, 227 gene sets were identified that were significantly different between malignant and benign tumors and were common between Ahus and METABRIC dataset. The genes within these pathways included mostly genes involved in interferon and cytokine signaling. The interferons are cytokines with important antineoplastic and immune modulatory effects involved in immune surveillance against cancer [21]. Through heatmaps generated with 227 immune pathways, we observed that the cancer samples were divided into two clusters with high and low pathway scores. The benign samples clustered among the cancer samples with low immunity. From these 227 pathways, five pathways were identified that could predict OS of breast cancer patients. The five pathways identified were part of many cellular processes, such as cytokine signaling, extracellular matrix degradation, and cellular responses to stress. However, one of the pathways had positive GSVA score, whereas four had negative GSVA score in malignant tumors compared to benign tumors. Previous research has identified that immune profiles of breast cancer patients can predict survival by dividing the patients into different immune clusters [20].

Furthermore, since immunity seemed to be involved in differences between malignant and benign tumors, we used deconvolution tools to identify the immune composition in the different tissues. Both DC and fTh cells were found to have significantly higher levels in benign tumors compared with malignant tumors. fTh cells are a subgroup of CD4+ T cells, which are required helpers of B‐cell maturation and antibody production with an important role in immune responses and autoimmune diseases [22]. In recent years, fTh cells have been characterized in several tumor entities in humans, and the infiltration of fTh cells was discovered to correlate positively with better prognosis in patients with malignant tumors, including breast cancer [23, 24, 25]. In the METABRIC dataset, we could not detect this significance, although, we do observe that fTh cells are higher in both malignant and benign tumors compared to normal tissue. There is one study published in which they looked at the presence of immune cells (CD45^+^) in breast tumor tissues compared with benign breast pathologies and found that total percentage of immune cells are higher expressed in benign compared to malignant tumors [26]. When looking into the different types of T lymphocytes in malignant and benign tumors, infiltrating helper T lymphocytes were increased in the case of malignant breast lesions, while cytotoxic T lymphocytes disclosed an opposite trend [26]. This is interesting results, and in line with our previous studies that suggest that infiltrating cytotoxic T lymphocytes are increased in the case of malignant breast lesions [15] while helper T lymphocytes are increased in colder tumors.

In the Ahus dataset, we also observe that macrophages are significantly different between many tissue types. Especially the M2 macrophages seem to be higher in normal tissue compared to benign and malignant tumors. There is increasing evidence that suggests that M2 macrophages could perform immunosuppressive functions and promote tumor progression and metastasis [27], and this is an area that needs to be further explored. The low number of benign tumors made the study vulnerable. One reason for this is that benign tumors are often classified as normal tissue, and therefore hard to identify when searching databases. The METABRIC dataset had a few benign tumors and was therefore considered as a good validation dataset. However, the benign tumors in METABRIC had different histology compared to the Ahus dataset. Whereas the Ahus dataset contained fibroadenomas and fibroadenomatosis, the METABRIC dataset contained mostly phyllodes tumors. This could explain some of the differences obtained when comparing results from both datasets, although studies have suggested they are highly related [28]. Furthermore, the study from Vidal et al. [9], showed that the overall gene expression profile of benign phylloides tumors was found more similar to fibroepithelial tumors and normal breast tissues than malignant tumors.

Nonetheless, the findings that benign tumors are different from malignant tumors regarding immune profiles is a novel finding that is worth to explore into more details. Although benign tumors rarely or never become cancerous, it is important to identify the molecular differences between the two tumor types, as this will lead us to better understanding of the tumor biology.

## 5 Conclusions

Using two different methods for pathway analysis (GSVA and Pathifier) and two independent datasets we identify immune‐related pathways as commonly significantly different (*P* < 0.05) between benign and malignant tumors, comprising of genes involved in interferon and cytokine signaling. Significantly higher abundance of resting dendritic cells (DC) and follicular T‐helper (fTh) was seen in benign tissue suggesting immune‐related pathways were distinctively different between benign and malignant tumors, and with different immune cell composition. Among those are GSE29614 and GSE360, highly correlated with CD4 memory resting T cells, memory B cells, Neutrophils, activated mast cells, activated natural killer cells, monocytes and M2 macrophages. Three other gene sets were highly correlated with gamma delta T cells, Naive B cells, M0 macrophages, resting and activated DC, Treg cells, fTh cells, CD4 naïve Tcells, resting NK cells, CD4 memory activated T cells and plasma cells. The findings that benign tumors are different from malignant tumors regarding immune profiles is a novel finding that is worth to explore into more details. Although benign tumors rarely or never become cancerous, it is important to identify the molecular differences between the two tumor types, as this will lead us to better understanding of the tumor biology.

## Conflict of interest

The authors declare no conflict of interest.

## Author contributions

VK, KS, AT, and LAT conceived the study idea, drafted, and edited the manuscript. XL, XT, SK, LAT, and AT performed the data analysis within the manuscript. MHR, TL, JG, LAT, and AT have been clinical Investigators and involved in obtaining patient material, clinical files, and provided constructive edits to the manuscript. All authors approved of the final version of the manuscript.

### Peer review

The peer review history for this article is available at https://www.webofscience.com/api/gateway/wos/peer‐review/10.1002/1878‐0261.13655.

## Supporting information


**Fig. S1.** Pathway deregulation scores across tissue types.

**Fig. S2.** Unsupervised heatmap of immune‐related pathways.

**Fig. S3.** Pathway scores and tissue clustering analysis.

**Fig. S4.** Expression of immune cell populations in tumor tissues.

**Table S1.** Clinical information for sample analysis.

**Table S2.** Gene sets differing between malignant and benign tumors.

**Table S3.** Significant pathways in METABRIC dataset.

**Table S4.** Comparison of gene sets in malignant and benign tumors.

**Table S5.** Overlapping pathways in Akershus and METABRIC datasets.

**Table S6.** Significantly different pathways in Akershus dataset.

**Table S7.** Pathway differences in tissue groups.

**Table S8.** Significant pathways in Akershus dataset.

**Table S9.** Pathway variations among tissue groups.

**Table S10.** Common pathways in Akershus and METABRIC datasets.

**Table S11.** Genes within specific pathways.

**Table S12.** Relevant pathways sorted by p‐value.

**Table S13.** Top pathways from GSE29614.

**Table S14.** Expression of immune cells in tumor tissues.

## Data Availability

All data are available either through previous publications, or as Supporting Information.
