# Supplementary figures and images for: Benign breast tumors may arise on different immunological backgrounds
Source: Mol Oncol. 2024 May 16;18(10):2495–509. doi: 10.1002/1878-0261.13655 (PMC11459044; doi:10.1002/1878-0261.13655)

## Pathway deregulation

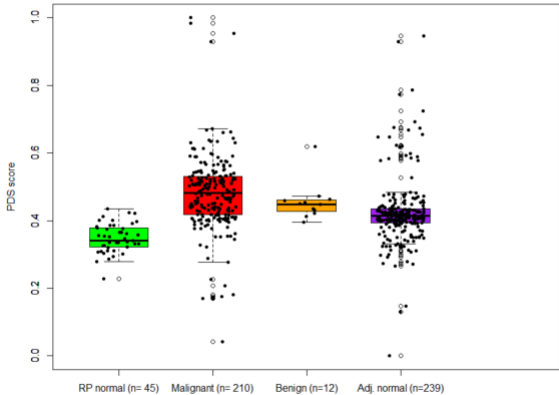

Supplement: Supplementary file 1 — Fig. S1. Pathway deregulation scores across tissue types. [file MOL2-18-2495-s016.pdf]

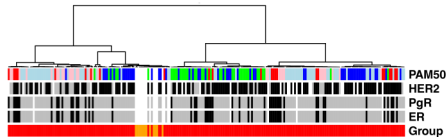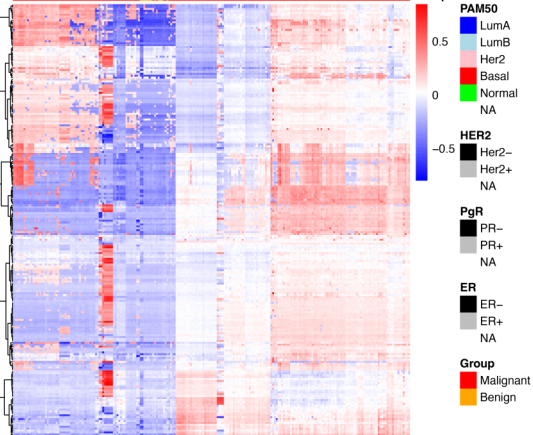

Supplement: Supplementary file 2 — Fig. S2. Unsupervised heatmap of immune‐related pathways. [file MOL2-18-2495-s015.pdf]

A

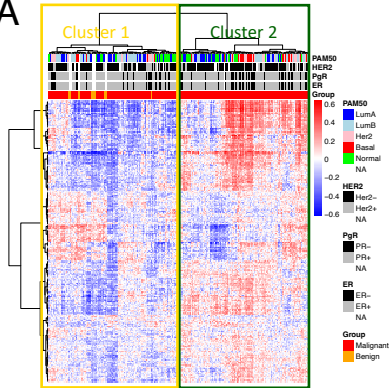

B

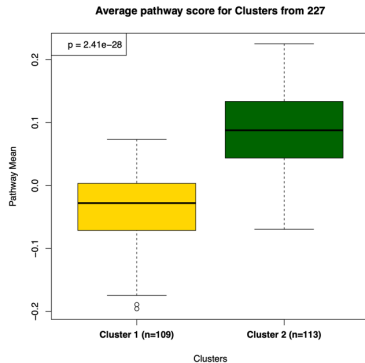

C

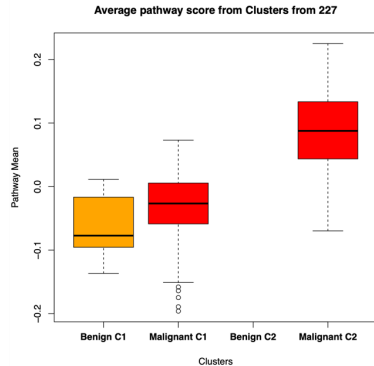

Supplement: Supplementary file 3 — Fig. S3. Pathway scores and tissue clustering analysis. [file MOL2-18-2495-s014.pdf]
