# Supplementary material for: Benign breast tumors may arise on different immunological backgrounds
Source: Mol Oncol. 2024 May 16;18(10):2495–509. doi: 10.1002/1878-0261.13655 (PMC11459044; doi:10.1002/1878-0261.13655)
Supplement: Supplementary file 4 — Fig. S4. Expression of immune cell populations in tumor tissues. [file MOL2-18-2495-s010.pdf]

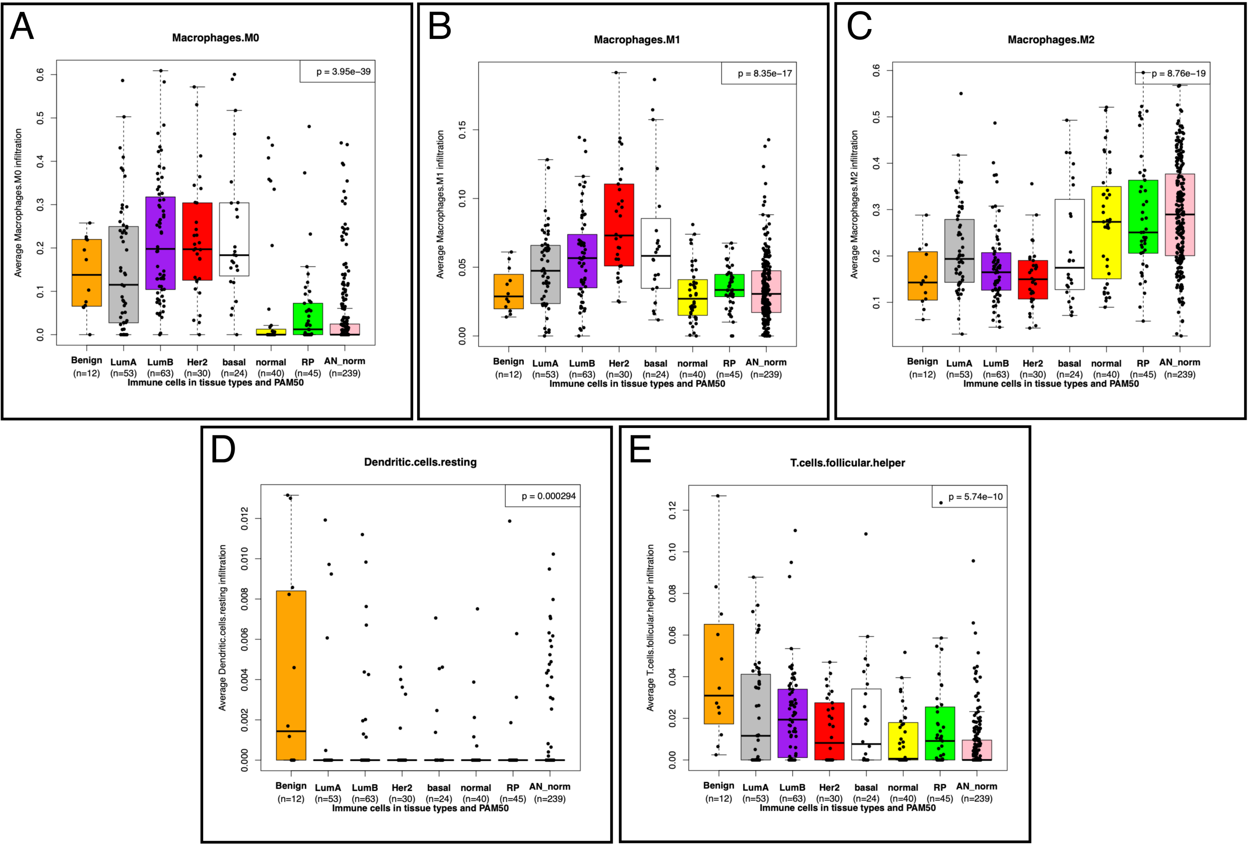
A
Macrophages.M0
p = 3.95e−39
Average Macrophages.M0 infiltration
Benign (n=12) LumA (n=53) LumB (n=63) Her2 (n=30) basal (n=24) normal (n=40) RP (n=45) AN_norm (n=239)
Immune cells in tissue types and PAM50
B
Macrophages.M1
p = 8.35e−17
Average Macrophages.M1 infiltration
Immune cells in tissue types and PAM50
C
Macrophages.M2
p = 8.76e−19
Average Macrophages.M2 infiltration
Immune cells in tissue types and PAM50
D
Dendritic.cells.resting
p = 0.000294
Average Dendritic.cells.resting infiltration
Immune cells in tissue types and PAM50
E
T.cells.follicular.helper
p = 5.74e−10
Average T.cells.follicular.helper infiltration
Immune cells in tissue types and PAM50
