# Supplementary material for: Benign breast tumors may arise on different immunological backgrounds
Source: Mol Oncol. 2024 May 16;18(10):2495–509. doi: 10.1002/1878-0261.13655 (PMC11459044; doi:10.1002/1878-0261.13655)
Supplement: Supplementary file 9 — Table S5. Overlapping pathways in Akershus and METABRIC datasets. [file MOL2-18-2495-s007.docx]

| **Genesets from MsigDB** | **Number of pathways of total pathways (n=)** | **% of total genesets** |
| --- | --- | --- |
| H hallmark gene sets | 1 (50) | 2% |
| c1 positional gene sets | 2 (300) | 0,6% |
| c2 curated gene sets | 239 (6290) | 31% |
| c3 regulatory target gene sets | 110 (3731) | 14% |
| c4 computational gene sets | 29 (858) | 4% |
| c5 ontology gene sets | 159 (14998) | 20% |
| c6 oncogenic signature gene sets | 3 (189) | 0,4% |
| **c7immune related** | **227 (4827)** | **29%** |
| c8 cell type signature gene sets | 6 (671) | 0,7% |
