# Supplementary material for: Benign breast tumors may arise on different immunological backgrounds
Source: Mol Oncol. 2024 May 16;18(10):2495–509. doi: 10.1002/1878-0261.13655 (PMC11459044; doi:10.1002/1878-0261.13655)
Supplement: Supplementary file 10 — Table S6. Significantly different pathways in Akershus dataset. [file MOL2-18-2495-s002.docx]

| **Pathifier – Ahus** | |
| --- | --- |
| Tissue types | Significant pathways |
| Tumor vs Benign | 19030 |
| Tumor vs Adjacent Normal | 26958 |
| Benign vs Adjacent Normal | 26896 |
| Benign vs Reduction mammoplasty | 20592 |
| Tumor vs Reduction mammoplasty | 28667 |
| Adjacent Normal vs Reduction mammoplasty | 25572 |
