# Supplementary material for: Benign breast tumors may arise on different immunological backgrounds
Source: Mol Oncol. 2024 May 16;18(10):2495–509. doi: 10.1002/1878-0261.13655 (PMC11459044; doi:10.1002/1878-0261.13655)
Supplement: Supplementary file 11 — Table S7. Pathway differences in tissue groups. [file MOL2-18-2495-s011.docx]

| **Function** | **MsigDB** | **T vs B**  **(n=)** | **T vs AjN**  **(n=)** | **B vs AjN**  **(n=)** | **RP vs T**  **(n=)** | **RP vs B**  **(n=)** | **RP vs AjN (n=)** |
| --- | --- | --- | --- | --- | --- | --- | --- |
| Hallmark gene sets | H hallmark gene sets | 28 | 46 | 45 | 48 | 32 | 38 |
| Positional gene sets | c1 positional gene sets | 149 | 212 | 215 | 224 | 177 | 215 |
| Curated gene sets | c2 curated gene sets | 3620 | 5379 | 5313 | 5690 | 4056 | 4897 |
| Regulatory target gene sets | c3 regulatory target gene sets | 2623 | 3107 | 3259 | 3327 | 2455 | 3292 |
| Computational gene sets | c4 computational gene sets | 521 | 668 | 751 | 799 | 528 | 691 |
| Ontology gene sets | c5 ontology gene sets | 8149 | 12124 | 11947 | 12905 | 8911 | 11532 |
| Oncogenic signature gene sets | c6 oncogenic signature gene sets | 70 | 174 | 175 | 182 | 160 | 115 |
| Immune related | c7immune related | 3399 | 4349 | 4362 | 4562 | 3538 | 4113 |
| Cell type signature gene sets | c8 cell type signature gene sets | 264 | 618 | 554 | 620 | 540 | 398 |
