# Supplementary material for: Benign breast tumors may arise on different immunological backgrounds
Source: Mol Oncol. 2024 May 16;18(10):2495–509. doi: 10.1002/1878-0261.13655 (PMC11459044; doi:10.1002/1878-0261.13655)
Supplement: Supplementary file 12 — Table S8. Significant pathways in Akershus dataset. [file MOL2-18-2495-s004.docx]

| GSVA Ahus | |
| --- | --- |
| **Tissue types** | **Significant pathways** |
| Tumor vs Benign | 12812 |
| Tumor vs Adjacent Normal | 24812 |
| Benign vs Adjacent Normal | 19137 |
| Benign vs Reduction mammoplasty | 13924 |
| Tumor vs Reduction mammoplasty | 21211 |
| Adjacent Normal vs Reduction mammoplasty | 29337 |
