# Supplementary material for: Benign breast tumors may arise on different immunological backgrounds
Source: Mol Oncol. 2024 May 16;18(10):2495–509. doi: 10.1002/1878-0261.13655 (PMC11459044; doi:10.1002/1878-0261.13655)
Supplement: Supplementary file 13 — Table S9. Pathway variations among tissue groups. [file MOL2-18-2495-s017.docx]

| **Function** | **MsigDB** | **T vs B**  **(n=)** | **T vs Adj.N**  **(n=)** | **B vs Adj. N**  **(n=)** | **B vs RP**  **(n)** | **T vs RP**  **(n=)** | **Adj.N**  **vs RP**  **(n=)** |
| --- | --- | --- | --- | --- | --- | --- | --- |
| Hallmark gene sets | H hallmark gene sets | 27 | 41 | 28 | 26 | 34 | 32 |
| Positional gene sets | c1 positional gene sets | 135 | 225 | 184 | 117 | 226 | 186 |
| Curated gene sets | c2 curated gene sets | 2625 | 4950 | 3813 | 2793 | 4197 | 3833 |
| Regulatory target gene sets | c3 regulatory target gene sets | 1254 | 2850 | 2185 | 1658 | 2392 | 2124 |
| Computational gene sets | c4 computational gene sets | 303 | 673 | 512 | 380 | 586 | 504 |
| Ontology gene sets | c5 ontology gene sets | 1798 | 3471 | 2720 | 1969 | 3048 | 2843 |
| Oncogenic signature gene sets | c6 oncogenic signature gene sets | 66 | 147 | 104 | 86 | 131 | 83 |
| Immune related | c7immune related | 2062 | 3873 | 2994 | 2138 | 3259 | 2938 |
| Cell type signature gene sets | c8 cell type signature gene sets | 135 | 239 | 181 | 131 | 202 | 190 |
