# Supplementary material for: Benign breast tumors may arise on different immunological backgrounds
Source: Mol Oncol. 2024 May 16;18(10):2495–509. doi: 10.1002/1878-0261.13655 (PMC11459044; doi:10.1002/1878-0261.13655)
Supplement: Supplementary file 14 — Table S10. Common pathways in Akershus and METABRIC datasets. [file MOL2-18-2495-s013.docx]

| **Function** | **MsigDB** | **Tumour VS Benign** |
| --- | --- | --- |
| Hallmark gene sets | H hallmark gene sets | 2 |
| Positional gene sets | c1 positional gene sets | 10 |
| Curated gene sets | c2 curated gene sets | 340 |
| Regulatory target gene sets | c3 regulatory target gene sets | 154 |
| Computational gene sets | c4 computational gene sets | 48 |
| Ontology gene sets | c5 ontology gene sets | 258 |
| Oncogenic signature gene sets | c6 oncogenic signature gene sets | 6 |
| Immune related | c7immune related | 306 |
| Cell type signature gene sets | c8 cell type signature gene sets | 11 |
