# Supplementary material for: Benign breast tumors may arise on different immunological backgrounds
Source: Mol Oncol. 2024 May 16;18(10):2495–509. doi: 10.1002/1878-0261.13655 (PMC11459044; doi:10.1002/1878-0261.13655)
Supplement: Supplementary file 16 — Table S12. Relevant pathways sorted by p‐value. [file MOL2-18-2495-s008.docx]

| **Pathway names** | **Entities** | | | | **Reactions** | |
| --- | --- | --- | --- | --- | --- | --- |
|  | **Found** | **Ratio** | **p-Value** | **FDR*** | **Found** | **Ratio** |
| Interferon alpha/beta signaling | 33/129 | 0.008 | 6.03e-10 | 9.73e-07 | 21/25 | 0.002 |
| Interferon signaling | 52/397 | 0.026 | 2.44e-05 | 0.02 | 54/119 | 0.008 |
| Cytokine signaling in immune system | 107/1100 | 0.071 | 5.78e-04 | 0.311 | 297/785 | 0.053 |
| Notch-HLH transcription pathway | 8/28 | 0.002 | 0.001 | 0.403 | 2/2 | 1.36e-04 |
| Ligand receptor interactions | 4/8 | 5.17e-04 | 0.003 | 0.733 | 4/4 | 2.72e-04 |
| Interferon gamma signaling | 23/177 | 0.011 | 0.005 | 0.733 | 3/23 | 0.002 |
| OAS antiviral response | 5/16 | 0.001 | 0.006 | 0.733 | 7/15 | 0.001 |
| BH3-only proteins associated within inactivated anti-apoptotic BCL-2 members | 4/11 | 7.11e-04 | 0.008 | 0.733 | 3/5 | 3.40e-04 |
| FOXO-mediated transcription of oxidative stress, metabolic and neuronal genes | 9/49 | 0.003 | 0.009 | 0.733 | 21/35 | 0.002 |
| Activation of NOXA and translocation to mitochondria | 3/6 | 3.88e-04 | 0.009 | 0.733 | 5/5 | 3.40e-04 |
| TRAF6 mediated IRF7 activation | 8/44 | 0.003 | 0.014 | 0.733 | 13/13 | 8.83e-04 |
| Negative regulators of DDX58/IFIH1 signaling | 7/37 | 0.002 | 0.017 | 0.733 | 11/13 | 8.83e-04 |
| NF-kB activation through FADD/RIP 1 pathway mediated by caspase-8 and -10 | 4/14 | 9.05e-04 | 0.018 | 0.733 | 5/5 | 3.40e-04 |
| LGI-ADAM interactions | 4/14 | 9.05e-04 | 0.018 | 0.733 | 3/5 | 3.40e-04 |
| Regulators of gene expression in early pancreatic percuser cells | 4/14 | 9.05e-04 | 0.018 | 0.733 | 2/6 | 4.08e-04 |
| DDX58/IFIH1- mediated induction of interferon alpha/beta | 14/105 | 0.007 | 0.019 | 0.733 | 43/53 | 0.004 |
| Transport of glycerol from adipocytes to the liver by Aquaporins | 2/3 | 1.94e-04 | 0.019 | 0.733 | 2/2 | 1.36e-04 |
| Cam-PDE 1 activation | 2/8 | 5.17e-04 | 0.02 | 0.733 | 2/22/2 | 1.36e-04 |
| TNFs binf their physiological receptors | 3/8 | 0.002 | 0.021 | 0.733 | 7/13 | 8.83e-04 |
| NOTCH1 Intercellular Domain Regulates Transcription | 6/30 | 0.004 | 0.022 | 0.733 | 13/18 | 0.001 |
| FOXO-mediated transcription | 14/110 | 0.007 | 0.027 | 0.733 | 58/85 | 0.006 |
| Myogenesis | 6/32 | 0.002 | 0.028 | 0.733 | 12/14 | 9.51e-04 |
| MECP2 regulates neuronal receptors and channels | 6/32 | 0.002 | 0.028 | 0.733 | 6/26 | 0.002 |
