# Supplementary material for: Benign breast tumors may arise on different immunological backgrounds
Source: Mol Oncol. 2024 May 16;18(10):2495–509. doi: 10.1002/1878-0261.13655 (PMC11459044; doi:10.1002/1878-0261.13655)
Supplement: Supplementary file 17 — Table S13. Top pathways from GSE29614. [file MOL2-18-2495-s003.docx]

| **Pathway names** | **Entities** | | | | **Reactions** | |
| --- | --- | --- | --- | --- | --- | --- |
|  | **Found** | **Ratio** | **p-Value** | **FDR*** | **Found** | **Ratio** |
| FGFR4 ligand binding and activation | 3/17 | 0.001 | 0.002 | 0.188 | 4/4 | 2.87e-04 |
| Phospholipase C mediated cascade; FGFR4 | 3/19 | 0.001 | 0.002 | 0.188 | 3/3 | 2.15e-04 |
| FGFR1 ligand binding and activation | 3/21 | 0.001 | 0.003 | 0.188 | 4/7 | 5.02e-04 |
| Phospholipase C mediated cascade; FGFR1 | 3/22 | 0.001 | 0.004 | 0.188 | 3/3 | 2.15e-04 |
| Activation point mutations of FGFR2 | 2/33 | 0.002 | 0.004 | 0.188 | 6/10 | 7.18e-04 |
| RUNX2 regulates chondrocyte maturation | 2/7 | 4.62e-04 | 0.004 | 0.188 | 2/4 | 2.87e-04 |
| Phospholipase C mediated cascade; FGFR2 | 3/25 | 0.002 | 0.005 | 0.1880 | 3/3 | 2.15e-04 |
| PI-3K cascafe:FGFR4 | 3/25 | 0.002 | 0.005 | 0.188 | 6/7 | 5.02e-04 |
| Ligand receptor interactions | 2/8 | 5.28e-04 | 0.006 | 0.188 | 4/4 | 2.87e-04 |
| Cam-PDE 1 activation | 2/8 | 5.28e-04 | 0.006 | 0.188 | 2/2 | 1.44e-04 |
