# Supplementary material for: Benign breast tumors may arise on different immunological backgrounds
Source: Mol Oncol. 2024 May 16;18(10):2495–509. doi: 10.1002/1878-0261.13655 (PMC11459044; doi:10.1002/1878-0261.13655)
Supplement: Supplementary file 18 — Table S14. Expression of immune cells in tumor tissues. [file MOL2-18-2495-s001.docx]

| **Celltype** | **Immune branch** | **Mean B** | **Mean M** | **Mean N** | **FDR B vs M** | **FDR B vs N** | **FDR M vs N** |
| --- | --- | --- | --- | --- | --- | --- | --- |
| B cells memory | adaptive | 0,031 | 0,017 | 0,026 | 2,6E-01 | 6,7E-01 | **1,9E-07** |
| B cells naive | adaptive | 0,008 | 0,012 | 0,004 | 7,5E-01 | 1,0E+00 | **3,5E-02** |
| Plasma cells | adaptive | 0,007 | 0,014 | 0,018 | 5,0E-01 | 6,2E-01 | 8,3E-01 |
| T-cells CD4 memory activated | adaptive | 0,041 | 0,037 | 0,005 | 4,3E-01 | **2,8E-05** | **7,1E-30** |
| T-cells CD4 memory resting | adaptive | 0,104 | 0,113 | 0,169 | 8,7E-01 | 1,1E-01 | **1,6E-12** |
| T-cells CD4 naive | adaptive | 0,000 | 0,006 | 0,005 | 4,9E-01 | 4,9E-01 | 9,7E-01 |
| T-cells CD8 | adaptive | 0,003 | 0,027 | 0,032 | 2,6E-01 | 1,5E-01 | 1,8E-01 |
| T-cells follicular helper | adaptive | 0,072 | 0,061 | 0,028 | 3,9E-01 | **4,6E-03** | **6,3E-18** |
| T-cells regulatory Tregs. | adaptive | 0,000 | 0,004 | 0,001 | 3,9E-01 | 5,4E-01 | **1,3E-03** |
| Dendritic cells activated | innate | 0,000 | 0,002 | 0,005 | 4,3E-01 | 1,5E-01 | **1,0E-07** |
| Dendritic cells resting | innate | 0,176 | 0,080 | 0,010 | 1,2E-01 | **3,3E-06** | **2,9E-15** |
| Eosinophils | innate | 0,000 | 0,000 | 0,000 | 7,6E-01 | NA | 1,5E-01 |
| M0 Macrophages | innate | 0,000 | 0,046 | 0,013 | 1,9E-01 | 3,8E-01 | **1,9E-07** |
| M1 Macrophages | innate | 0,074 | 0,044 | 0,028 | 1,3E-01 | **1,9E-03** | **2,1E-11** |
| M2 Macrophages | innate | 0,212 | 0,286 | 0,433 | 2,6E-01 | **2,1E-04** | **2,6E-38** |
| Mast cells activated | innate | 0,019 | 0,022 | 0,052 | 7,5E-01 | 1,5E-01 | **4,9E-10** |
| Mast cells resting | innate | 0,160 | 0,062 | 0,028 | 1,6E-01 | **4,9E-03** | **1,6E-06** |
| Monocytes | innate | 0,010 | 0,040 | 0,027 | 4,3E-01 | 3,0E-01 | 9,2E-01 |
| Neutrophils | innate | 0,004 | 0,002 | 0,003 | 4,9E-01 | 1,7E-01 | **7,6E-03** |
| NK cells activated | innate | 0,000 | 0,010 | 0,044 | 2,6E-01 | **1,9E-03** | **7,1E-40** |
| NK cells resting | innate | 0,066 | 0,039 | 0,006 | 1,6E-01 | **3,3E-06** | **5,1E-14** |
| T-cells gamma delta | innate | 0,013 | 0,078 | 0,065 | 1,6E-01 | **1,3E-02** | 3,8E-01 |
